# Supplementary material for: Streamlines of the Poynting Vector and Chirality Flux around a Plasmonic Bowtie Nanoantenna
Source: Nanomaterials (Basel). 2023 Dec 25;14(1):61. doi: 10.3390/nano14010061 (PMC10781037; doi:10.3390/nano14010061)
Supplement: Supplementary file 1 [file nanomaterials-14-00061-s001.zip › nanomaterials-2730615-supplementary.pdf]

Supplementary Materials

Streamlines of the Poynting Vector and Chirality Flux around a Plasmonic Bowtie Nanoantenna

Yun-Cheng Ku [1,2], Mao-Kuen Kuo [2,*] and Jiunn-Woei Liaw [1,3,4,*]

1 Department of Mechanical Engineering, Chang Gung University, 259 Wen-Hwa 1st Rd., Kwei-Shan, Taoyuan 333, Taiwan; d05543005@ntu.edu.tw
2 Institute of Applied Mechanics, National Taiwan University, 1, Sec. 4, Roosevelt Rd., Taipei 106, Taiwan
3 Department of Mechanical Engineering, Ming Chi University of Technology, New Taipei City 24301, Taiwan
4 Proton and Radiation Therapy Center, Linkou Chang Gung Memorial Hospital, Taoyuan 333423, Taiwan
* Correspondence: mkkuo@ntu.edu.tw (M.-K.K.); markliaw@mail.cgu.edu.tw (J.-W.L.)

Recently, we developed a numerical method of boundary element method (BEM) combined with method of moment (MoM) for the simulation of electromagnetic field [1]. This method can be applied to study the light-matter interaction of not only multiple scattereres or nanoparticles (NPs) but also multiple multi-layered scatterers. In the following, various plasmonic nanostructures are simulated. The permittivity of Au and Ag is referred to Ref. [2]. The surrounding medium is water with a refractive index of 1.33. To verify the accuracy of BEM/MoM, we also used Mie theory [3] and (MMP) method for simulation [4].

1. Coreshell

A spherical core-shelled NP is analyzed to demonstrate the numerical convergence. The core is Au NP with a radius of 50 nm coated with a TiO_2 shell (refractive index of 2.1) of thickness of 100 nm, immersed in water. The efficiencies of scattering cross section (SCS) and absorption cross section (ACS) of the coreshell, irradiated by a linearly polarized (LP) plane wave, are shown in Figure S1, where the results calculated by Mie theory and BEM/MoM are plotted, respectively. The numbers of the surface triangular mesh for BEM/MoM are 864 for the discretization of the outer interface (water/TiO_2) and the inner interface (TiO_2/Au), respectively. Both results are consistent. However, the accuracy of BEM/MoM at the short-wavelength region can be improved by raising the number of triangular mesh.

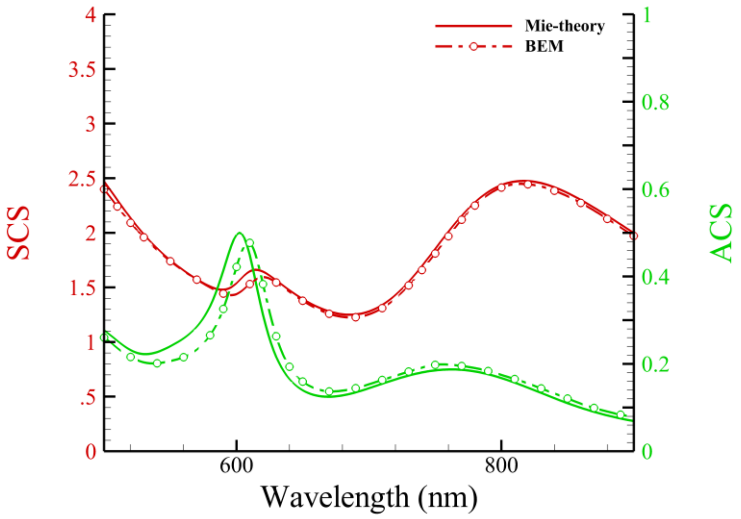

Figure S1. Spectra of efficiencies of SCS and ACS of a coreshell, Au NP coated with TiO_2 layer, in water, irradiated by a LP plane wave. The radius of Au NP is 50 nm, and the thickness of TiO_2 layer is 100 nm. Solid line: Mie theory. Dash line: BEM/MoM. Mesh number is 864 at the inner and outer interfaces, respectively.

To study the local energy confinement and the streamline of energy flux (Poynting vector) around a coreshell, we used MMP method. For example, Ag NP (r= 70 nm) is the core coated with a Si layer (thickness: 160 nm) irradiated by a x-polarized plane wave upward propagating along z axis [5]. The wavelength-dependent dielectric constant of Si is referred to Ref. [6]. Figure S2a shows the spectra of efficiencies of SCS and ACS of coreshell in water. The electromagnetic distributions in nearfield at two peaks of ACS, λ= 938 nm and 1187 nm, are analyzed. Figures S2b and S2c show the maps of streamlines of energy flux and $|\mathbf{E}|^2$ in the x-z plane and y-z plane cross sections, respectively, at λ= 938 nm. Figures S2d and S2e show the results in the x-z plane and y-z plane cross sections, respectively, at λ= 1187 nm. The local energy confinement and the intricate vortex of energy-flux streamline are observed in these figures.

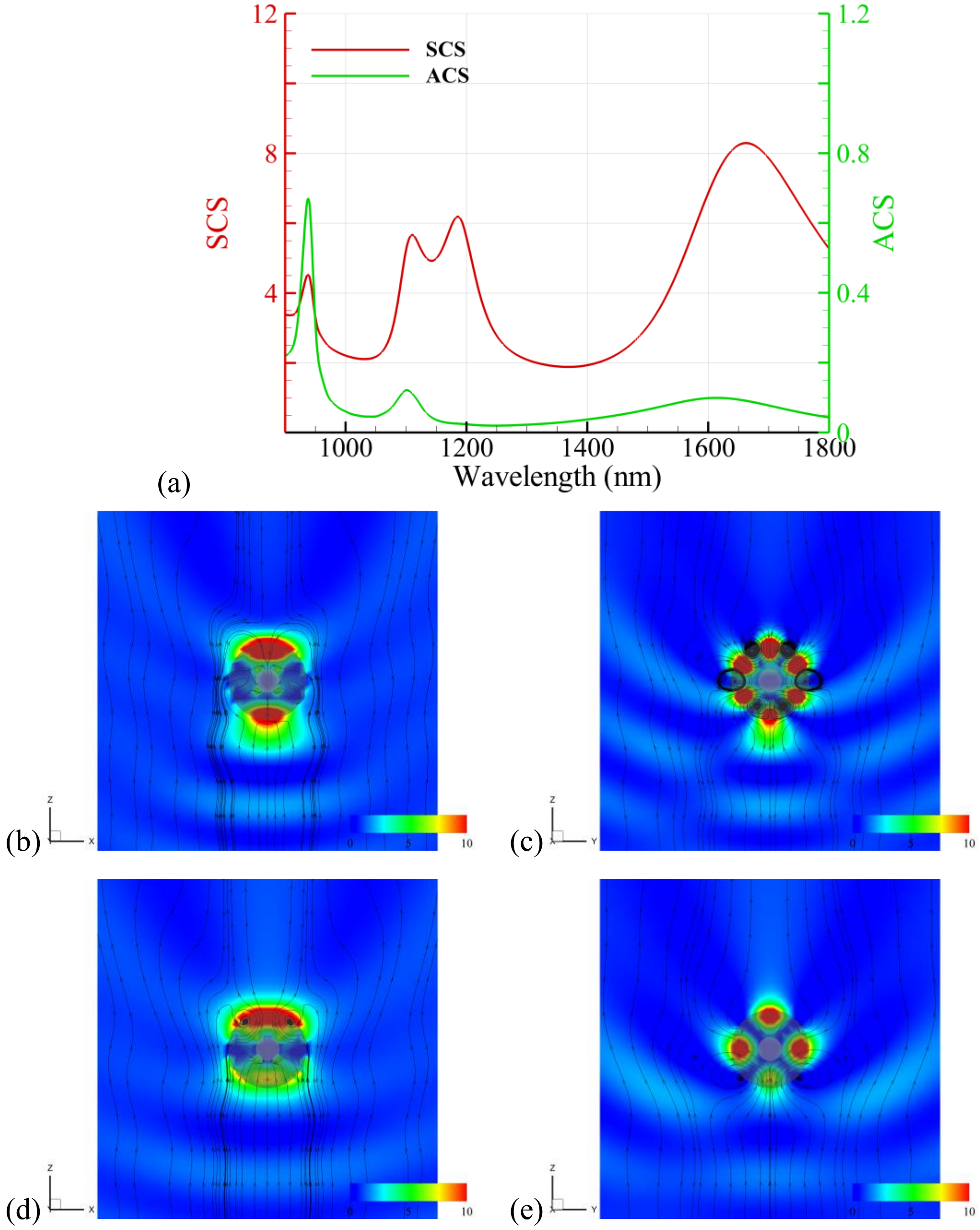

Figure S2. (a) Spectra of efficiencies of SCS and ACS of a coreshell in water, irradiated by a x-polarized plane wave upward propagating along z axis; Ag NP of r= 70 nm is coated with Si layer of 160-nm thickness. Maps of streamlines of energy flux and $|\mathbf{E}|^2$ of (b) x-z plane and (c) y-z plane cross sections at λ= 938 nm. Maps of streamlines of energy flux and $|\mathbf{E}|^2$ of (d) x-z plane and (e) y-z plane cross sections at λ= 1187 nm. Color bar: $|\mathbf{E}|^2$.

2. Dimer

We used BEM/MoM to the light-matter interaction of a plasmonic homodimer, a pair of identical Au NPs placed along x axis with a gap of 10 nm, in water. The radius of Au NPs is 50 nm. The spectra of efficiencies of SCS and ACS of Au dimer irradiated by x-polarized and y-

polarized incident plane wave propagating along z axis are plotted in Figure S3, respectively. The surface mesh number is 864 for each NP.

Another case of a heterogeneous dimer, consisting of Au NP and Si NP, with a gap of 30 nm, is also studied. The radius of Au NP and Si NP is 50 nm. The surrounding medium is water. The dielectric constants of Au and Si are referred to Refs. [2, 6]. The illuminating plane wave is circularized polarized (CP). The results of BEM/MoM with 484 meshes on each NP and MMP are shown in Figure S4. Both results are in agreement.

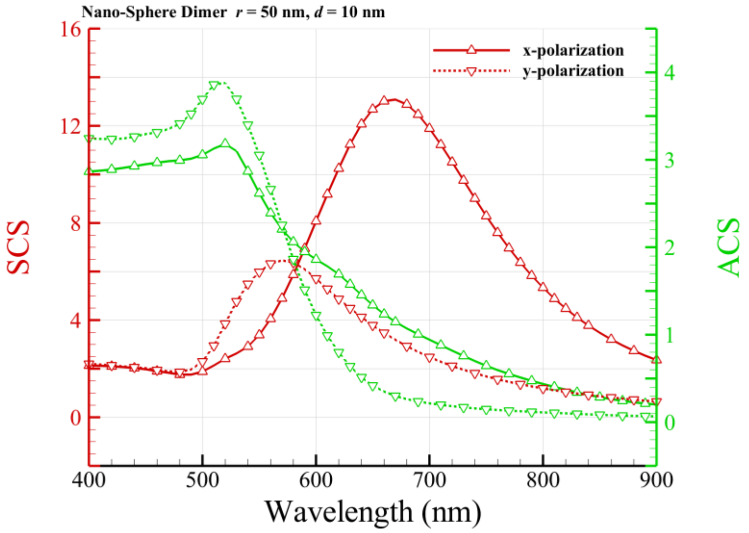

Figure S3. Spectra of efficiencies of SCS and ACS of a homodimer, a pair of identical Au NPs along x axis, in water. The radius of Au NPs is 50 nm and the gap is 10 nm. Δ: x-polarized and ∇: y-polarized incident plane waves. These results are calculated by BEM/MoM. Surface mesh number is 864 for each NP.

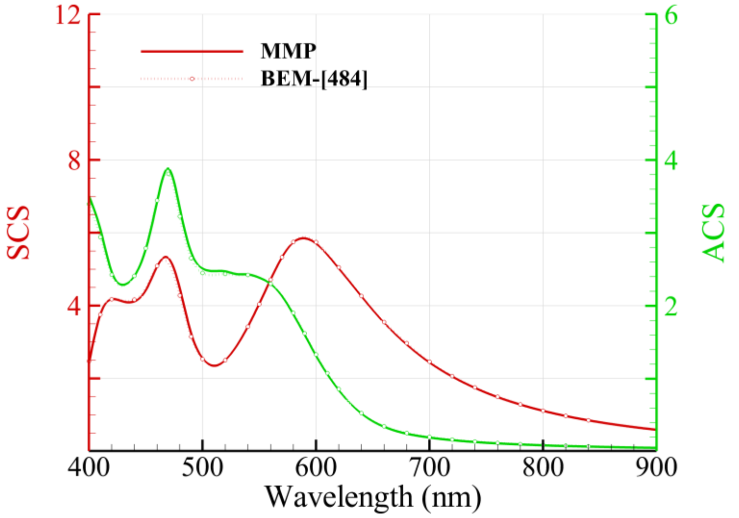

Figure S4. Spectra of efficiencies of SCS and ACS of a heterogeneous dimer, Au NP and Si NP along x axis, irradiated by CP plane wave in water. The radii of these NPs is 50 nm and the gap

is 30 nm. Solid line: MMP. Circle symbol: BEM/MoM with 484 meshes on each NP.

3. Bowtie nanoantenna

The top-view distributions of electric field and streamlines of energy flux around Au bowtie nanoantenna (side length: 100 nm, thickness: 10 nm, round corner: 3 nm), irradiated by an upward propagating right-handed (RH) CP plane wave at λ= 870 nm, is shown in Figure S5.

Figure S6 shows the spectra of efficiencies of SCS and ACS of Ag bowtie nanoantenna in photon energy. For a single Ag nanotriangle in water, the peak of the ACS spectrum (black) is at 1.55 eV (λ= 800 nm), as shown in the following Figure. The Rabi splitting energies of Ag bowtie nanoantenna are at 1.48 eV (λ= 780 nm) and 1.59 eV (λ= 840 nm). The distributions of electric field and energy flux around Ag bowtie nanoantenna (side length: 100 nm, thickness: 10 nm, round corner: 3 nm), irradiated by a perpendicular-polarized plane wave and parallel-polarized plane wave of λ= 633 nm, respectively, are shown in Figure S7.

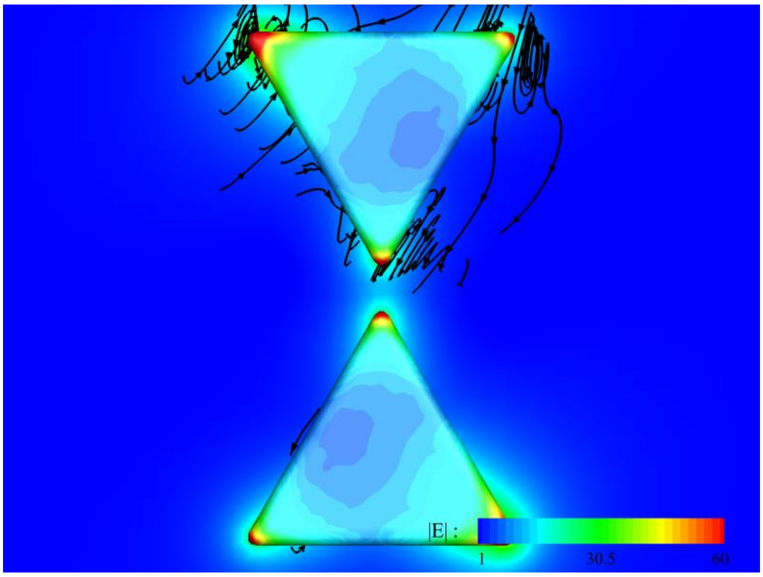

Figure S5. Distributions of electric field (color) and streamlines of energy flux (black) around Au bowtie nanoantenna (side length: 100 nm, thickness: 10 nm, round corner: 3 nm) at λ= 870 nm (the top view).

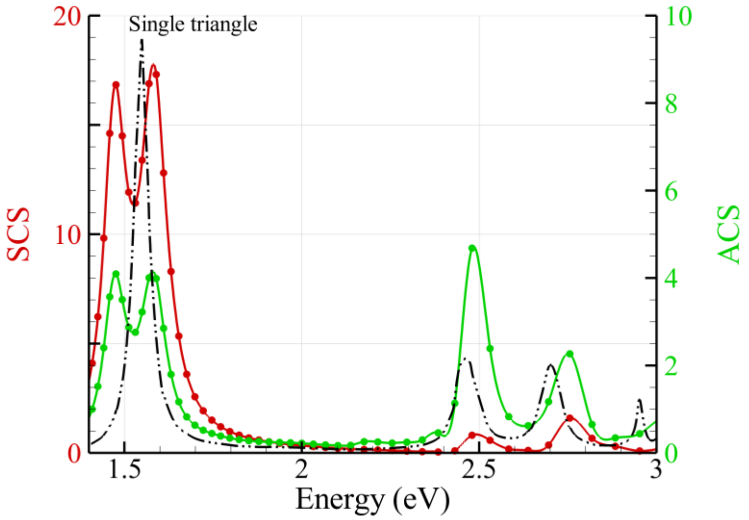

Figure S6. Spectra of efficiencies of SCS and ACS of Ag bowtie nanoantenna (side length: 100 nm, thickness: 10 nm, round corner: 3 nm) in photon energy, as irradiated by an upward propagating RH CP plane wave. The peak in ACS of a single Ag nanotriangle (black) is at 1.55 eV (λ= 800 nm).

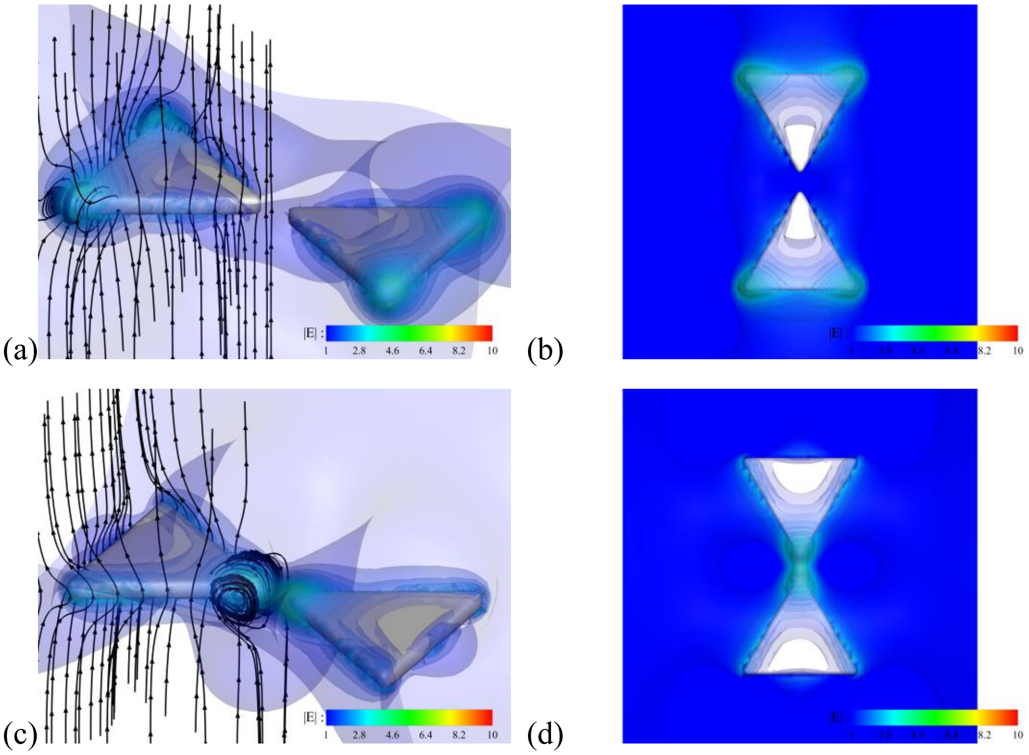

Figure S7. Distributions of electric field (blue) and streamlines of energy flux (black) around Ag bowtie nanoantenna (side length: 100 nm, thickness: 10 nm, round corner: 3 nm), irradiated by a (a) & (b) perpendicular-polarized plane wave and (c) & (d) parallel-polarized plane wave of λ= 633 nm, respectively. (b) & (d): top view.

References

1. Ku, Y. C.; Kuo, M. K.; Liaw, J. W. Winding Poynting vector of light around plasmonic nanostructure. *J. Quant. Spectrosc. Radiat. Transfer* **2022**, *278*, 108005.
2. Johnson, P. B.; Christy, R. W. Optical constants of the noble metals. *Phys. Rev. B* **1972**, *6*, 4370–4379.
3. Liaw, J. W.; Chen, H. C.; Kuo, M. K. Plasmonic Fano resonance and dip of Au-SiO2-Au nanomatryoshka. *Nanoscale Res Lett.* **2013**, *8*, 468.
4. Liaw, J. W.; Tsai, H. Y. Theoretical investigation of plasmonic enhancement of silica-coated gold nanorod on molecular fluorescence. *J. Quant. Spectrosc. Radiat. Transfer* **2012**, *113*, 470−479.
5. Barreda, Á. I; Gutiérrez, Y.; Sanz, J. M.; González, F.; Moreno, F. Polarimetric response of magnetodielectric core–shell nanoparticles: an analysis of scattering directionality and sensing. *Nanotechnology* **2016**, *27*, 234002.
6. Palik, E. D. *Handbook of Optical Constants of Solids*, Academic Press, Boston, **1985**. https://www.filmetrics.com/refractive-index-database/Si/Silicon
